# Maternal Vitamin D Prevents Abnormal Dopaminergic Development and Function in a Mouse Model of Prenatal Immune Activation

**DOI:** 10.1038/s41598-018-28090-w

**Published:** 2018-06-27

**Authors:** Wei Luan, Luke Alexander Hammond, Stephanie Vuillermot, Urs Meyer, Darryl Walter Eyles

**Affiliations:** 1 Queensland Brain Institute, The University of Queensland, Brisbane, QLD Australia; 2 Zuckerman Mind Brain Behavior Institute, Columbia University, New York, New York USA; 3 Physiology and Behavior Laboratory, ETH Zurich, Schwerzenbach, Switzerland; 4 Institute of Pharmacology and Toxicology, University of Zurich-Vetsuisse, Zurich, Switzerland; 5 Neuroscience Centre Zurich, University of Zurich and ETH Zurich, Zurich, Switzerland; 6 Queensland Centre for Mental Health Research, Brisbane, QLD Australia

## Abstract

Dysfunction in dopamine (DA) systems is a prominent feature in schizophrenia patients and may result from the abnormal development of mesencephalic (mes)DA systems. Maternal immune activation (MIA) and developmental vitamin D (DVD)-deficiency both induce schizophrenia-relevant dopaminergic abnormalities in adult offspring. In this study, we investigated whether maternal administration of the vitamin D hormone (1,25OHD, VIT_D_) could prevent MIA-induced abnormalities in DA-related behaviors and mesDA development. We administrated the viral mimetic polyriboinosinic-polyribocytidylic (poly (I:C)) simultaneously with 1,25OHD and/or their vehicles, to pregnant mouse dams at gestational day 9. Maternal treatment with VIT_D_ prevented MIA-induced hypersensitivity to acute DA stimulation induced by amphetamine, whereas it failed to block prepulse inhibition deficiency in MIA-exposed offspring. MIA and VIT_D_ both reduced fetal mesDA progenitor (Lmx1a + Sox2+) cells, while VIT_D_ treatment increased the number of mature (Nurr1 + TH+) mesDA neurons. Single-cell quantification of protein expression showed that VIT_D_ treatment increased the expression of Lmx1a, Nurr1 and TH in individual mesDA cells and restored normal mesDA positioning. Our data demonstrate that VIT_D_ prevents abnormal dopaminergic phenotypes in MIA offspring possibly via its early neuroprotective actions on fetal mesDA neurons. Maternal supplementation with the dietary form of vitamin D, cholecalciferol may become a valuable strategy for the prevention of MIA-induced neurodevelopmental abnormalities.

## Introduction

Today schizophrenia is conceptualized as a polygenetic disorder possibly triggered by diverse environmental factors. Epidemiological studies implicate a growing number of pre- and perinatal environmental risk factors, including fetal hypoxia, obstetric complications, maternal infection and/or immune activation (MIA), and certain nutritional deficiencies such as developmental vitamin D (DVD)-deficiency^1–4^. These factors combine to affect the normal trajectory of brain development in ways that remain opaque.

Dopamine dysregulation has long been proposed as an important pathophysiological feature of schizophrenia. Molecular imaging studies suggest that neurochemical abnormalities of presynaptic dopamine (DA) uptake and/or release may be core to the psychotic component in most schizophrenic patients^5^. We have proposed that early alterations in the trajectory of developing DA neurons may represent a first step in the journey towards the dopaminergic changes observed in schizophrenia^6^.

Animal models of MIA and DVD-deficiency provide support for this hypothesis^7–10^. Not only do these models capture DA-related behavioral and cognitive deficits relevant to schizophrenia^7–14^, but also they suggest that dopaminergic alterations originate in early fetal development^14–16^. DA neurons are born very early around 5 weeks post conception (1^st^ trimester) in the human embryonic mesencephalon^17^, which is roughly equivalent to gestational day (GD) 9.5 in mice^18,19^. MIA on GD 9 affects the genesis of mesencephalic dopamine (mesDA) neurons and affects the expression of genes crucial for their establishment, including sonic hedgehog (SHH) and fibroblast growth factor 8 (FGF8)^14^. MIA also changes the fetal expression of nuclear receptor related 1 protein (Nurr1), which is essential for mesDA differentiation and maintenance^11,14^. In DVD-deficient rat fetuses, the ratio of DA neurons between the two major mesDA neuronal subgroups (the substantia nigra pars compacta (SNc) and ventral tegmental area (VTA)), are altered^16^. DVD-deficiency in rats also reduces the expression of Nurr1 and tyrosine hydroxylase (TH), the rate-limiting enzyme in DA synthesis in mesDAs^16,20–22^. MIA and DVD-deficiency in rodents also lead to altered DA metabolisms in neonatal brains^13,23–25^.

MIA is also a risk factor for other psychiatric conditions most noticeably autism^11,26^. Using a MIA model that is based on maternal treatment with the viral mimetic polyriboinosinic-polyribocytidylic (poly (I:C)), we have recently shown that when the active vitamin D hormone (1,25OHD) (here on referred to as VIT_D_) is administered to MIA-exposed dams simultaneously with poly(I:C), it prevents certain MIA-induced behavioral deficits in associative learning, stereotyped digging and social interaction in juvenile offspring^27^. Such behavioural disruptions are consistent but not exclusive to autism-related phenotypes. In the present study, we investigated whether administration of VIT_D_ to MIA-exposed dams also prevents DA-related behavioral alterations in MIA offspring. We further explored the effects of VIT_D_ and MIA on the expression of factors involved in early mesDA neuron maturation. To this aim, we established an automated image analysis method for the identification and measurement of individual mesDA cells. This method allowed us to assess the acute effects of MIA and VIT_D_ treatments on mesDA neurogenesis and maturation at the single-cell level. GD11 was chosen to examine post-MIA effects at this time point, which allowed us to assess both active mesDA neurogenesis and the early stages of mesDA neuron maturation. We show that although MIA and VIT_D_ both reduced mesDA progenitor numbers, VIT_D_ treatment increased mature mesDA neuron number and increased the expression of key mesDA differentiation factors. Therefore, one plausible neuroprotective action of VIT_D_ may be its pro-differentiating role in mesDA neurogenesis in the MIA-exposed fetuses.

## Materials and Methods

### Animals

Female and male breeder C57BL6/N mice were obtained from Charles River Laboratories (Sulzfeld, Germany) at the age of 12–14 weeks. They were kept in regular open cage systems (Macrolon Type-III, Turnhout, Belgium) and had had a libitum access to water and standard rodent chow (Kliba 3430, Kaiseraugst, Switzerland) that contained 1000IU cholecalciferol/kg. Breeding began after 2 weeks of acclimatization to the animal holding rooms. Animals were housed and tested under a reversed light–dark cycle (lights on from 19h00 to 07h00). All procedures described in the present study had been approved by the Cantonal Veterinarian’s Office of Zurich, and all efforts were made to minimize the number of animals used and their suffering.

### Experimental groups

The active hormonal form of VIT_D_ (1α, 25-dihydroxy vitamin D3 = 1,25OHD; solid powder, Calbiochem, EMD Millipore, Cat No 679101-50UG, Switzerland) was prepared as described previously^27^. Sterile corn oil (Sigma–Aldrich, Switzerland) was used as vehicle (VEH). Poly(I:C) (potassium salt; Sigma–Aldrich, Switzerland) was prepared using sterile, pyrogen-free 0.9% NaCl (saline), and injected as described previously^28^. Saline (CON) was used as the vehicle for poly (I:C).

C57BL6/N female mice were subjected to a timed-mating procedure as described previously^28^. Pregnant dams on GD 9 were first injected subcutaneously with VIT_D_ (400 ng/kg/2 ml) or vehicle (VEH), and then immediately injected intravenously with either poly(I:C) (POL, 5 mg/kg; calculated based on the pure form) or saline solution (CON) at a volume of 5 ml/kg as described previously^27,28^. Thus, the experiment consisted of 4 groups: CON-VEH, CON-VIT_D_, POL-VEH, POL-VIT_D_. VIT_D_ dose was chosen based on pilot studies that showed no effects on AMPH-mediated locomotion or PPI (Fig. S1).

### Behavioral Studies

Weaned offspring were housed in same-sex groups of 3–4 animals per cage^28^. Behavioral testing was conducted at 10 weeks of age. To minimize possible confounds arising from litter effects^29^, only 1 male offspring per litter was randomly selected. Hence, the number of offspring in each experimental group was equal to the number of treated dams. The group sizes were as follows: *N*(CON-VEH) = 11, *N*(CON-VIT_D_) = 8, *N*(POL-VEH) = 11, and *N*(POL-VIT_D_) = 8. All offspring were first subjected to prepulse inhibition (PPI), followed by amphetamine (AMPH) locomotion two to three days later. All behavioral tests were performed in the dark phase of the light-dark cycle.

### Prepulse Inhibition of the Acoustic Startle Reflex

PPI was conducted using startle chambers (San Diego Instruments, California, USA) as described previously (for details see suppl methods)^27,28^. PPI was indexed by percentage inhibition of the startle response obtained in pulse-alone trials by the following expression: 100% × (1 − [mean reactivity on prepulse-plus- pulse trials/mean reactivity on pulse-alone trials]). In addition, the animals’ reactivity to pulse-alone trials (i.e., startle reactivity) and prepulse-alone trials (i.e., prepulse-induced reactivity) were also measured and analyzed.

### Spontaneous and Amphetamine-induced Locomotor Activity

Testing conditions, lighting and amphetamine dose were all as previously described^11,28^. Briefly, the animals were first acclimatized to the open field for 20 min and were then injected with sterile 0.9% saline and immediately returned to the same arenas for another 20 min. Subsequently, the animals were injected intraperitoneally (i.p.) with D-amphetamine sulfate (=AMPH; Sigma-Aldrich, Switzerland) at dose of 2.5 mg/kg (5 ml/kg) and monitored for a period of 60 min.

### Fetal Tissue Collection and Immunohistochemistry

A second cohort of pregnant dams were prepared and assigned to poly(I:C), VIT_D_, or appropriate vehicle on GD 9 as described above. The number of dams assigned to each treatment group was *N*(CON-VEH) = 5, *N*(CON-VIT_D_) = 4, *N*(POL-VEH) = 5, and *N*(POL-VIT_D_) = 7. Two fetuses per dam were randomly collected (see below), so that the number of fetus in each group was *n*(CON-VEH) = 10, *n*(CON-VIT_D_) = 8, *N*(POL-VEH) = 10, and *N*(POL-VIT_D_) = 14.

Fetal mesDA development was assessed at GD11 for the following reasons. First, at this age, the four major mesDA subpopulations we wished to assess (see below) are all present^30^. Second, by GD 11 the elevation in maternal inflammatory cytokines and chemokines induced by MIA has returned to baseline levels^31–33^, thus removing any active cytokine-mediated cellular effects. Third, the half-life of 1,25OHD is 4–6 hours^34^. Therefore, it is likely the active agent would have been eliminated after 48 hrs.

To collect the fetuses, dams were decapitated without prior anesthesia. The uterus was exposed and collected fetuses were immersion-fixed in 4% paraformaldehyde overnight. Fixed brains were soaked in 30% sucrose solution prior to embedding (Tissue-Tek, Emgrid, Australia). The mesencephalon cryosections were obtained in a one in three series. Sections were immunohistochemically processed simultaneously to eliminate variability. One series was processed for Sox2/Lmx1a, and a second series for Nurr1/TH. Primary antibodies were: rabbit anti-Sox2 (1:200, Millipore Chemicon, Australia), goat anti-Lmx1a (1:100, Santa Cruz Biotechnology, USA), sheep anti-TH (1:100, Novus Biologicals, USA), and rabbit anti-Nurr1 (1:500, Santa Cruz Biotechnology, USA). Fluorophore-conjugated secondary antibodies were diluted 1:1000 (Thermo Fisher Scientific, Australia). Nuclei were stained using 4′,6-diamidino-2-phenylindole (DAPI, 1:1000, Sigma-Aldrich, Australia).

### Image acquisition and quantitative immunochemistry

Immunostaining was assessed using a confocal microscope (Tie, Nikon Inc.) equipped with a Spectral Applied Research’s Diskovery spinning disk module with an inverted spinning disk. Images were captured and stitched using NIS software (Nikon Inc.). Images were acquired using 60x oil N.A. 1.4 objective (CFI Apo Lamda/W.D. 0.14 mm), providing a pixel size 0.092 × 0.092 µm^2^. Four mesDA subpopulations were assessed: mesDA progenitors (Lmx1a+Sox2+), post-mitotic (Lmx1a+Sox2−) mesDA neurons, immature (Nurr1+TH−) mesDA neurons, and mature (Nurr1+TH+) mesDA neurons. Briefly, images were background-corrected using ImageJ software and were then analyzed using CellProfiler software (Broad Institute, Massachusetts, USA). This allowed us to quantify cell number, mediolateral/dorsoventral positions, individual protein expression (mean gray value per cell), and nuclear morphological parameters (for image processing and quantitative immunochemistry details see Fig. S2).

### Statistical Analysis

All data were analyzed using parametric analysis of variance (ANOVA). Percent PPI was analyzed using a 2 × 2 × 3 × 3 (POL treatment × VIT_D_ treatment × pulse level × prepulse level) ANOVA. In the AMPH sensitivity test, the total distance moved was expressed as a function of 5-min bins and was analyzed by 2 × 2 × 4 (POL treatment × VIT_D_ treatment × 5-min bins) repeated-measure ANOVAs for the initial habituation and saline-treatment phases, and by 2 × 2 × 12 (POL treatment × VIT_D_ treatment × 5-min bins) repeated-measure ANOVA for the subsequent AMPH treatment phase. The quantitative immunochemistry data were analyzed using a 2 × 2 (POL treatment × VIT_D_ treatment) ANOVA using a repeated-measure design (repeated for section intervals). The distribution data were analyzed using a 2 × 2 (POL treatment × VIT_D_ treatment) ANOVA using a repeated-measure design (repeated for bins). Following these initial ANOVAs, restricted ANOVAs or Dunnett’s Multiple Comparison Tests were conducted whenever appropriate. Statistical significance was set at *p* < 0.05.

## Results

### Effect of maternal VITD treatment on MIA-induced behaviors

**Figure 1:**
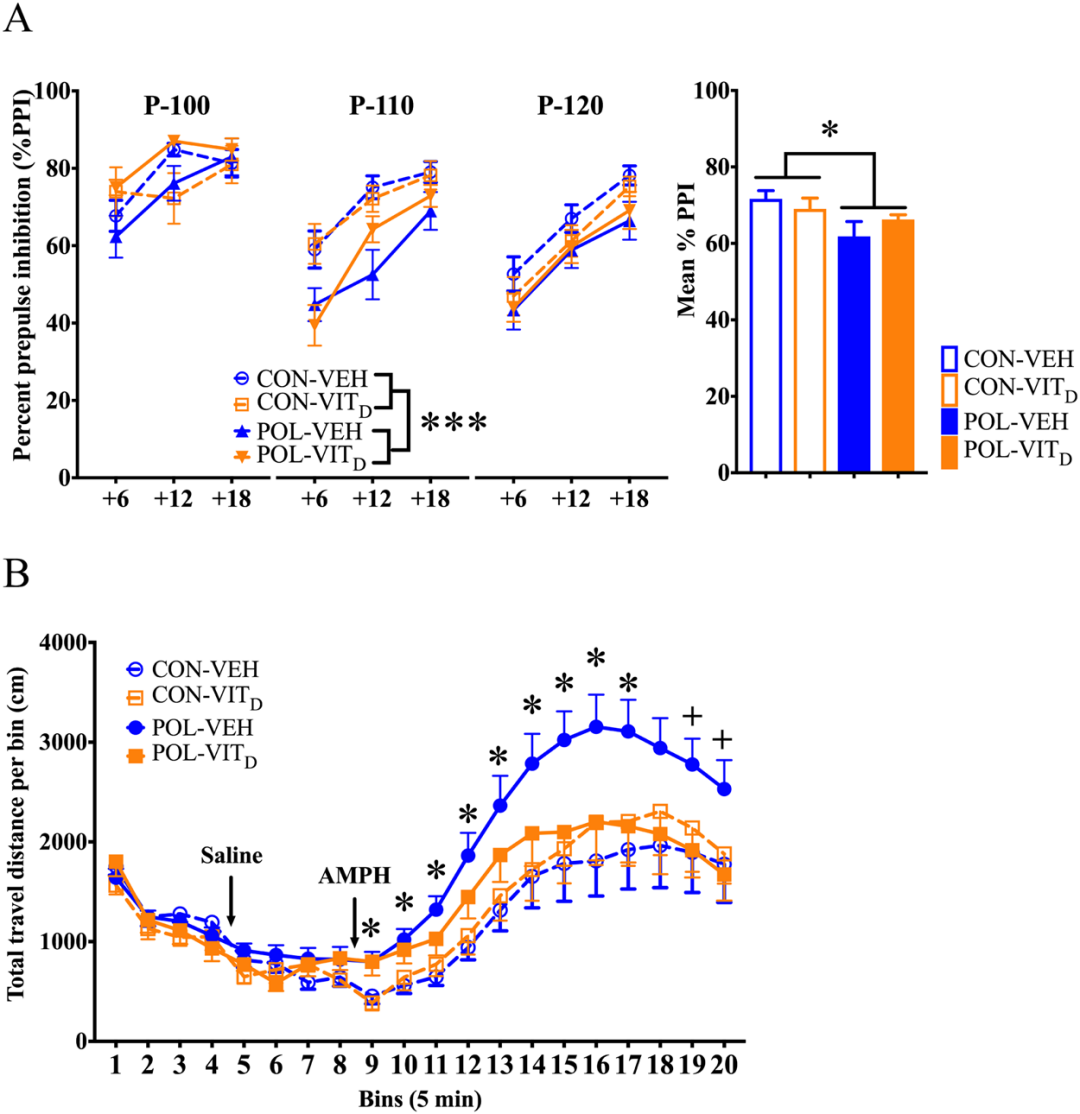
The effects of single and combined MIA and VIT_D_ on prepulse inhibition and amphetamine sensitivity in adult offspring. (**A**) Prepulse inhibition (PPI) of the acoustic startle reflex was used to investigate sensorimotor gating in adult offspring. %PPI is shown for three startling amplitudes (100, 110 and 120 dB_A_, which are noted as P-100, P-110 and P-120, respectively) and prepulse intensities (71, 77 and 83 dB_A_, corresponding to +6, +12 and +18 dB_A_ above background white noise). Poly(I:C) exposure impaired P-110dB_A_ PPI. The bar plots represent the mean %PPI for all prepulse and pulse levels. All values were medians ±SEM. ^*^p < 0.05, reflecting the significant main effect of MIA on mean % PPI; ^***^p < 0.001, reflecting the significant main effect of MIA in the P-110 condition. (**B**) The line plot shows total distance travelled in an open field per bin (=5 min) during initial habituation and subsequent saline administration periods, followed by the amphetamine (AMPH; 2.5 mg/kg, i.p.) administration phase. ^*^p < 0.05, reflecting the significant difference between POL-VEH and CON-VEH offspring (AMPH bins 1–9); ^+^*p* < 0.05, reflecting the significant difference between POL-VEH and POL-Vit_D_ offspring (AMPH bins 11 and 12). All values were means ± SEM.

Consistent with previous studies^14,28,32^, MIA impaired PPI in adult offspring (main overall effect of MIA: *F*_(1,34)_ = 4.63, *p* < 0.05; Fig. 1A). The MIA-induced effect on PPI was driven primarily by a marked reduction the 110 dB_A_ pulse condition (main effect of MIA treatment at the 110 dB_A_ condition: *F*_(1,34)_ = 13.19, *p* < 0.001), but not in the 100 or 120 dB_A_ conditions (100 dB_A_: *F*_(1,34)_ = 0.67, *p* = 0.42; 120 dB_A_: *F*_(1,34)_ = 3.04, *p* = 0.09). Maternal VIT_D_ treatment did not prevent MIA-induced PPI impairments (Fig. 1A).

In contrast, maternal VIT_D_ treatment prevented the emergence of AMPH hypersensitivity in offspring exposed to MIA. In line with previous findings^14,23,28,32^, AMPH-induced locomotor activity was increased in MIA offspring (Fig. 1B). This effect of MIA was blocked when pregnant dams were co-treated with VIT_D_, leading to a significant three-way interaction between POL treatment, VIT_D_ treatment, and bins (*F*_(11,34)_ = 1.83, *p* < 0.05). Subsequent post-hoc group comparisons at each individual 5-min bin confirmed a significant increase in POL-VEH mediated locomotion relative to CON-VEH offspring (all p < 0.05; Fig. 1B), and between POL-VEH and POL-Vit_D_ offspring at bin 11 and 12 (*p’s* < 0.05; Fig. 1B). Neither MIA nor maternal VIT_D_ treatment affected locomotor activity in the initial habituation and saline treatment phases of the test (Fig. 1B).

### MIA and VITD treatments both reduce mesDA progenitor number

**Figure 2:**
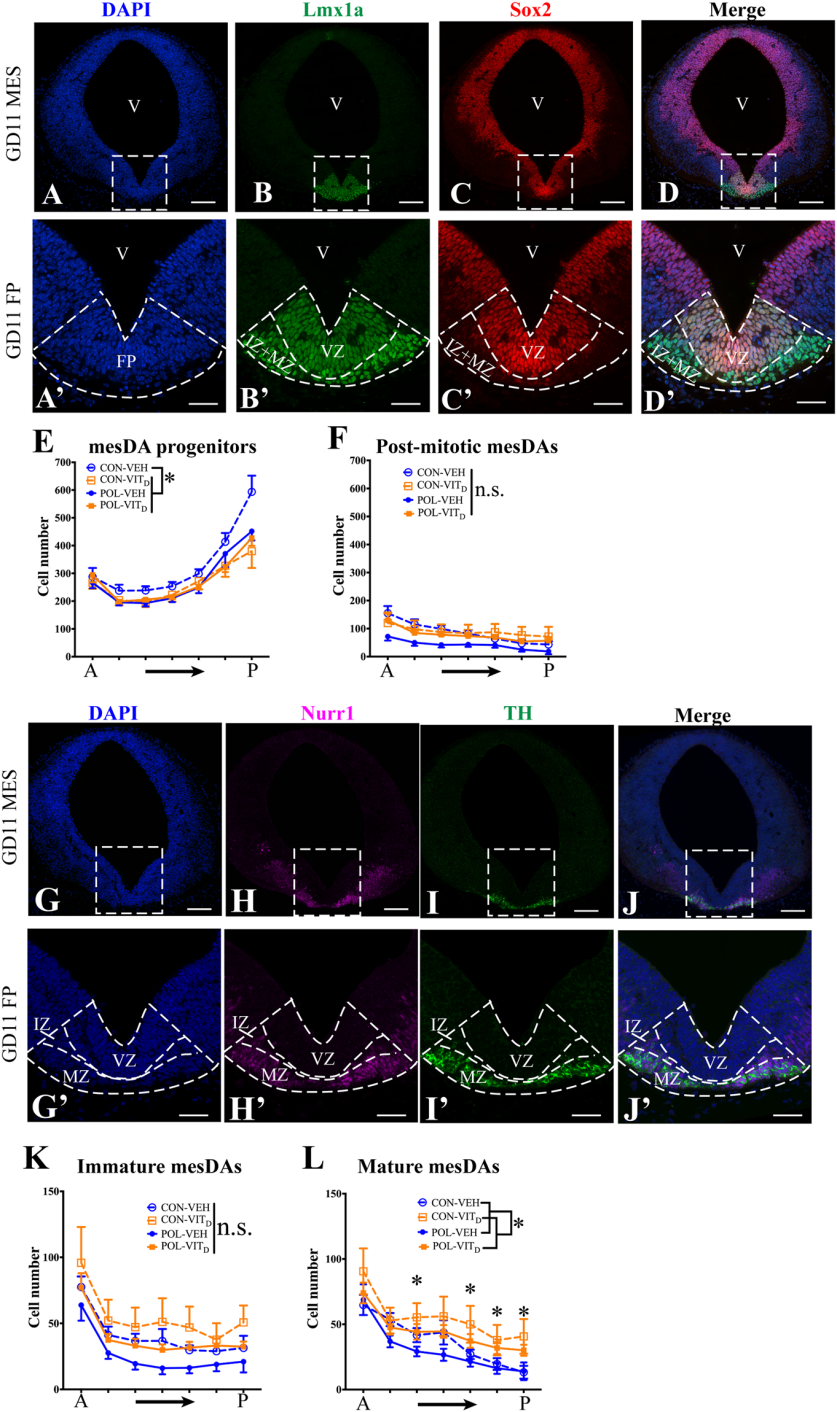
The effects of single and combined MIA and VIT_D_ on early mesencephalic dopamine (mesDA) cell number. (**A**–**D**) Fate mapping of developing mesDA cells in a medial coronal mesencephalic (MES) sections at gestational day (GD) 11. Markers are the nuclear dye DAPI (blue, A), mesDA specification factor Lmx1a (green, B), neuronal progenitor maker Sox2 (red, C), and a channel-merged image (**D**). (A’–D’) Higher magnification images of the dashed outlined boxes representing the floor plate (FP) from (**A**–**D**) accordingly. Lmx1a specifically marked mesDA cells in the FP dorsoventrally from ventricular zone (VZ), intermediate zone (IZ) to mantle zone (MZ). MesDA progenitors (Lmx1a+Sox2+) were primarily located in the proliferative VZ, in contrast, post-mitotic (Lmx1a+Sox2−) mesDAs were mostly located in IZ and MZ. (**E**) The cell number of these two mesDA subpopulations were counted using CellProfiler software at 60 µm intervals along the anterior-posterior (A–P) axis. The numbers of mesDA progenitors (Lmx1a+Sox2+) were reduced by all treatments relative to the control (CON-VEH) (*p’s* < 0.05). (**F**) Post-mitotic (Lmx1a+Sox2−) mesDA number was not altered by any treatment. (**G**–**J**) Fate mapping of post-mitotic mesDA subgroups that are characterized by Nurr1 and TH in medial coronal MES sections at GD11. Markers are the nuclear dye DAPI (blue, **G**), Nurr1 (magenta, H), TH (green, I), and a channel-merged image (J). (G’–J’) Higher magnification images of the dashed outlined boxes representing FP from (**G**–**J**) accordingly. Immature Nurr1+ mesDAs were primarily located in the IZ and MZ. Mature (Nurr1+ TH+) mesDAs were primarily located in MZ. (**K**) There were no significant differences of immature mesDA number among treatment groups (*p’s* > 0.05). (**L**) VIT_D_ treatment itself increased mature (Nurr1+ TH+) mesDA number compared to its vehicle (VEH) (*p* < 0.05). VIT_D_ treatment itself (CON-VIT_D_) particularly increased mature mesDA number in the posterior MES compared to control (CON-VEH) (*p’s* < 0.05). All values were means ± SEM. ^*^P < 0.05. n.s., represents not statistically significant. Scale bars: 100 µm (A–D and G–J); 50 µm (A’–D’ and G’–J’).

At GD11, mesDA progenitors are still proliferating^18,19^. Here we examined mesDA progenitors co-expressing the mesDA specification factor Lmx1a and the neural stem cell transcription factor Sox2 (Fig. 2A–D and A’–D’). Sox2 is absent in post-mitotic Lmx1a + mesDAs (Fig. 2C–D and C’–D’). There was a significant interaction between MIA and maternal VIT_D_ treatment on the number of mesDA progenitors (Lmx1a+Sox2 +) (*F*_(1,36)_ = 5.19, *p* < 0.05; Fig. 2E). Post hoc comparisons revealed that mesDA progenitors were significantly reduced in MIA-treated fetuses (POL-VEH) (*F*_(1,36)_ = 6.68, *p* < 0.05), VIT_D_-treated fetuses (CON-VIT_D_) (*F*_(1,36)_ = 8.12, *p* < 0.05) and MIA-VIT_D_ co-treated fetuses (POL-VIT_D_) (*F*_(1,36)_ = 4.51, *p* < 0.05) relative to controls (CON-VEH). Hence, MIA and VIT_D_ both reduced mesDA progenitor numbers in GD11 fetuses.

### Maternal VITD treatment increases the number of mature mesDAs

Consistent with previous studies^18^, immature (Nurr1+TH−) cells were found in the IZ, whereas mature (Nurr1+TH+) cells were largely restricted to the MZ (Fig. 2G–J and G’-J’). MIA and VIT_D_ had no effect on the number of immature mesDAs (*p*’s > 0.05; Fig. 2K). However, there was a significant main effect of maternal VIT_D_ treatment on mature mesDAs (*F*_(1,40)_ = 5.574, *p* < 0.05; Fig. 2L). Significant interactions of MIA x VIT_D_ treatments with section position was also found along the anterior-posterior (A-P) axis of the MES (*F*_(6,240)_ = 2.941, *p* < 0.05). Post hoc comparison revealed that VIT_D_ treatment itself (CON-VIT_D_) increased mature mesDA number relative to control (CON-VEH), particularly in the posterior MES (*p’s* < 0.05).

### MIA and VITD treatments affect the position of mesDA progenitors and mature mesDAs

**Figure 3:**
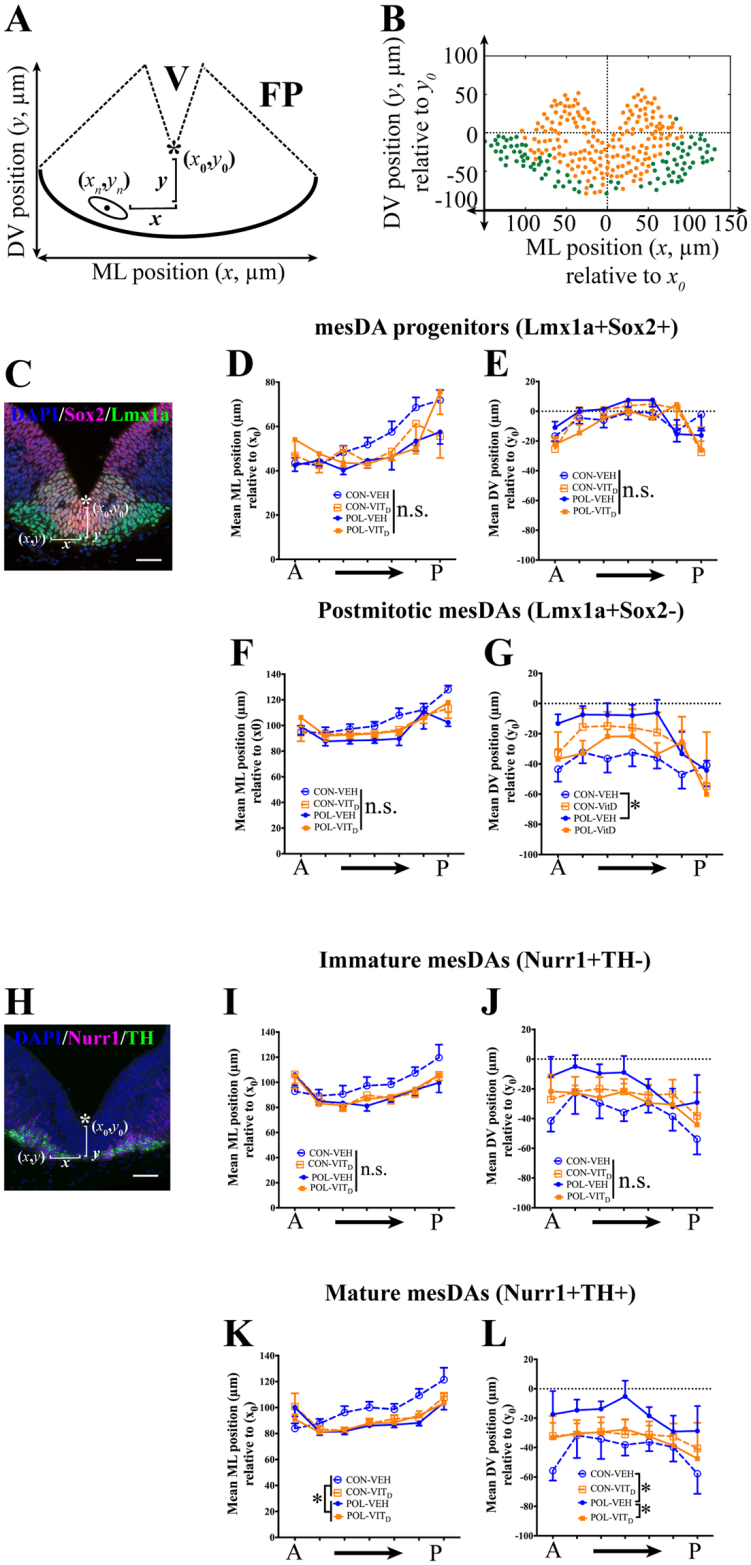
The effects of single and combined MIA and VIT_D_ treatments on the coronal positioning of mature mesencephalic dopamine neurons (mesDAs). (**A**) Schematic image of registered floor plate (FP) in a medial coronal mesencephalic (MES) section with mediolateral (ML, *x*) and dorsoventral (DV, *y*) positions of cells. The coordinates (*x*_0_, *y*_0_) were chosen as the most ventral point of ventricle (V) along the midline (*). The black dot represents the mean center positioning (*x*_*n*_, *y*_*n*_) of a representative mesDA nucleus (ellipse). The ML positioning (*x*) of mesDA cells was measured bilaterally as the absolute distance from the center of mesDA nucleus (*x*_*n*_) to the coordinate (*x*_0_) = *ABS* (*x*_*n*_*−x*_0_). The DV positioning (*y*) of mesDA cell was calculated as the distance from the center of mesDA nucleus (*y*_*n*_) to the coordinate (*y*_0_) = (*y*_0_−*y*_*n*_). (**B**) An example of mesDA positioning in the FP. Yellow dots represent mesDA progenitors (Lmx1a+Sox2+), and green dots represent post-mitotic (Lmx1a+Sox2−) mesDAs. (**C**) A representative FP section showing cells that were triple-labeled by DAPI (blue), Lmx1a (green) and Sox2 (red). No significant effects of MIA or VIT_D_ treatment were noticeable for the mean ML (**D**) or DV (**E**) positioning of mesDA progenitors (*p’s* > 0.05). (**F**) There were no significant effects of MIA or VIT_D_ treatments on the mean ML positioning of post-mitotic (Lmx1a+Sox2−) mesDAs (*p’s* > 0.05). (**G**) There were significant interactions between MIA or VIT_D_ treatments on the average DV positioning of post-mitotic (Lmx1a+Sox2−) (*p* < 0.05). MIA treatment (POL-VEH) reduced the DV positioning of post-mitotic mesDAs compared to its control (CON-VEH) (*p* < 0.05). (**H**) A representative FP of a coronal MES section showing cells that were triple-labeled by DAPI (blue), Nurr1 (magenta) and TH (green). There were no significant effects of MIA or VIT_D_ treatment for the mean ML (**I**) or DV (**J**) positioning of immature (Nurr1+TH−) mesDAs in the FP (*p’s* > 0.05). (**K**) MIA treatment (POL) reduced the mean ML positioning of mesDAs relative to CON (*p* < 0.05). (**L**) There were significant interactions between MIA x VIT_D_ treatments in the average DV positioning of mature (Nurr1+TH+) mesDAs (*p* < 0.05). Post hoc comparison revealed MIA treatment (POL-VEH) decreased the mean DV positioning relative to the control (CON-VEH) (*p* < 0.05). Additionally, the co-treatment of VIT_D_ restored the mean DV positioning of mature mesDAs in POL-VIT_D_ group compared to POL-VEH group (*p* < 0.05). All values were means ± SEM. ^*^p < 0.05. n.s. represents not statistically significant. Scale bars: 50 µm.

CellProfiler allowed us to position individual mesDAs in the floor plate (FP). We assessed the average mediolateral (ML) position, (*x* distance from the midline of the coronal MES section, *x*_0_) and average dorsoventral (DV) position (*y* distance from the most ventral point of the ventricle, *y*_0_) (Fig. 3A,B).

MIA or VIT_D_ had no effect on the ML or DV positioning of mesDA progenitors (Lmx1a + Sox2+) in the FP (*p’s* > 0.05; Fig. 3C–E). There were also no significant effects of MIA or VIT_D_ treatments on the average ML positioning of post-mitotic (Lmx1a + Sox2−) mesDAs (*p’s* > 0.05; Fig. 3F). There was significant interactions between MIA and VIT_D_ treatments on the average DV positioning of post-mitotic (Lmx1a + Sox2−) (*F*_(1,36)_ = 4.616, *p* < 0.05; Fig. 3G). Subsequent post hoc comparison revealed that MIA treatment (POL-VEH) reduced the DV positioning of post-mitotic mesDAs compared to its control (CON-VEH) (*F*_(1,36)_ = 5.997, *p* < 0.05).

There were no significant main effects or interactions of MIA or VIT_D_ treatments on the average ML or DV positioning of immature (Nurr1 + TH−) mesDAs in the FP (*p’s* > 0.05; Fig. 3H–J). In contrast, there was a significant main effect of MIA treatment on the mean ML positioning of mature (Nurr1+TH+) mesDAs (*F*_(1,40)_ = 5.962, *p* < 0.05; Fig. 3K). In addition, there were significant interactions between MIA x VIT_D_ treatments in the average DV positioning of mature (Nurr1+TH+) mesDAs (*F*_(1,40)_ = 5.128, *p* < 0.05; Fig. 3L). Post hoc comparisons showed that MIA treatment (POL-VEH) reduced the DV positioning of mature mesDAs compared to its control (CON-VEH) (*F*_(1,40)_ = 5.871, *p* < 0.05). Most intriguingly, abnormal DV positioning of mature mesDAs in MIA fetuses was restored by the co-administration of VIT_D_ (POL-VIT_D_) (*F*_(1,40)_ = 6.125, *p* < 0.05).

### VITD treatment increases the expression of Lmx1a, Nurr1 and TH proteins in mesDAs

mesDA proliferation, specification and differentiation are tightly regulated by the transcription factors Sox2, Lmx1a and Nurr1^18,19^. CellProfiler analysis allowed us to quantify the protein expression of these factors in individual mesDA cells by measuring the mean fluorescence intensity of these factors.

#### Sox2 expression

**Figure 4:**
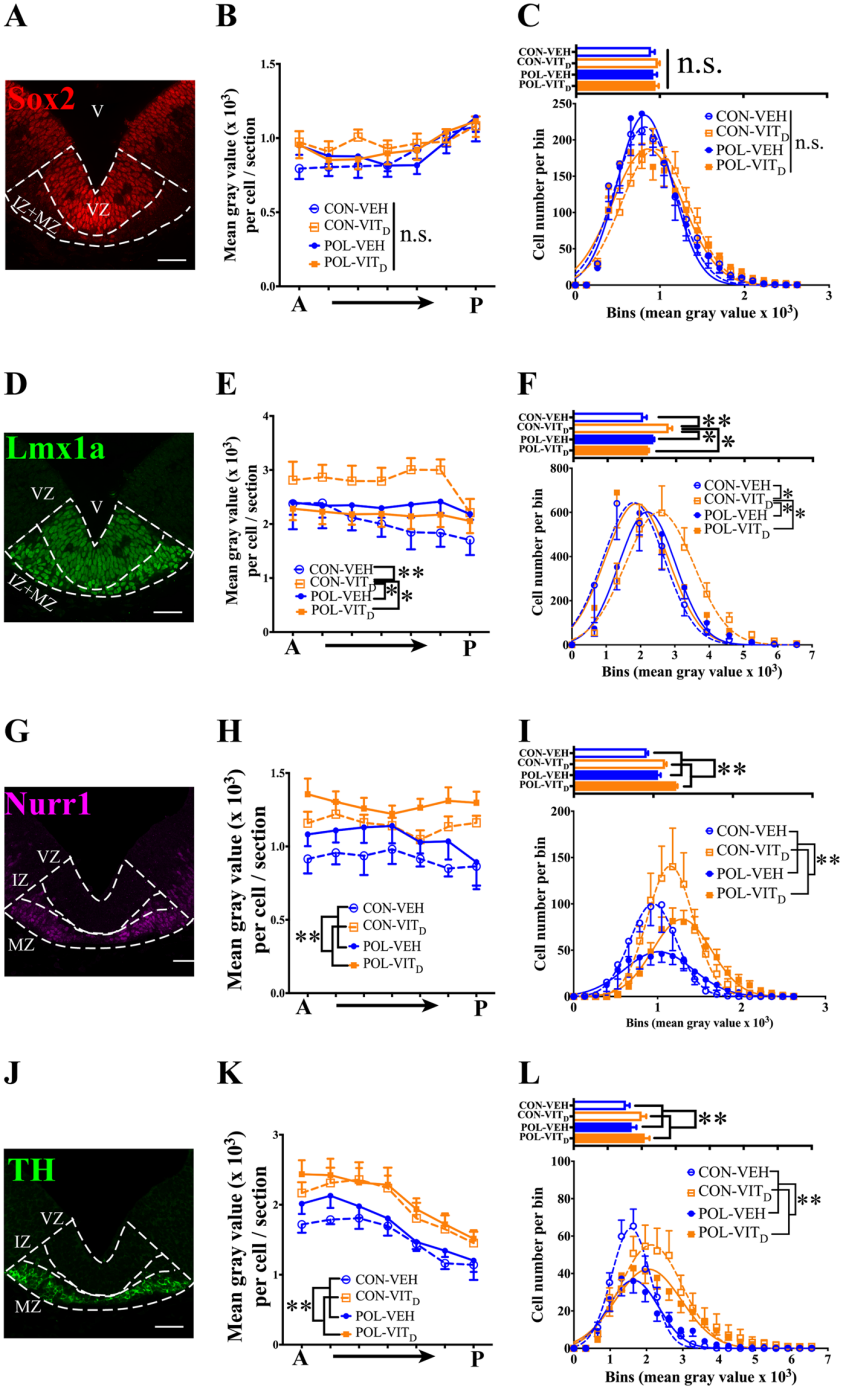
The effects of single and combined MIA and VIT_D_ treatment on the expression of factors important in the development of mesDA neurons. (**A**) A representative floor plate (FP) of coronal MES section showing mesDA cells expressing Sox2 (red). (**B**) Neither MIA or VIT_D_ treatment altered the mean intensity of Sox2 in mesDA progenitors (Lmx1a+Sox2+) (*p’s* > 0.05). (**C**) Bar plots represent the average fluorescence of Sox2 in mesDA progenitors among treatment groups. Distribution analysis representing a nonlinear regression to predict the Gaussian distribution of mesDA progenitor number relative to the intensity of Sox2 expression. (**D**) A representative FP of coronal MES section showing post-mitotic mesDA cells expressing Lmx1a (green). (**E**) VIT_D_ treatment significantly increased the mean intensity of Lmx1a in mesDAs compared with other treatment groups (*p’s* < 0.05). (**F**) Bar plots represent the average expression of Lmx1a among treatment groups. Distribution analysis showed VIT_D_ treatment itself increased the number of cells expressing higher levels of Lmx1a compared to other treatment groups (*p’s* < 0.05). (**G**) A representative FP of coronal MES section showing post-mitotic mesDA cells expressing Nurr1 (magenta). (**H**) VIT_D_ treatment significantly increased the expression of Nurr1 in post-mitotic (Nurr1+) mesDAs (*p* < 0.05). (**I**) Bar plots represent the average fluorescence of Nurr1 among treatment groups. Distribution analysis showed VIT_D_ treatment increased the number of cells expressing higher levels of Nurr1 compared to VEH (*p* < 0.05). (**J**) A representative FP of coronal MES section showing mature mesDAs expressing TH (green). (**K**) VIT_D_ treatment increased the average fluorescence of TH in mature (Nurr1+TH+) mesDAs. (**L**) Bar plots represent the average TH mean intensity in mature mesDAs among treatment groups. The distribution analysis verified that VIT_D_ treatment increased the number of mature (Nurr1+TH+) mesDAs that expressed higher levels of TH compared to VEH (*p* < 0.01). All values were means ± SEM. ^*^p < 0.05, ^**^p < 0.01. Scale bars: 50 µm.

MIA and VIT_D_ had no effect on the mean intensity of Sox2 fluorescence in individual mesDA progenitors (*p’s* > 0.05; Fig. 4B). Distribution analysis also confirmed Sox2 staining was constant across all treatments (*p’s* > 0.05; Fig. 4C).

#### Lmx1a expression

There was a significant main effect of VIT_D_ treatment on the mean intensity of Lmx1a expression (*F*_(1,36)_ = 5.891, *p* < 0.05), and significant interactions between MIA x VIT_D_ treatments (*F*_(1,36)_ = 7.993, p < 0.05; Fig. 4E). Post hoc comparisons revealed that Lmx1a expression was significantly higher in individual cells of CON- VIT_D_ fetuses compared to CON-VEH (*p* < 0.01) and the other two treatment groups (*p’s* < 0.05). Supporting this, the distribution analysis of Lmx 1a+ demonstrated that more CON-VIT_D_ mesDA cells expressed higher levels of Lmx1a compared to other treatment groups (*p’s* < 0.05; Fig. 4F).

#### Nurr1 expression

There was a significant main effect of VIT_D_ treatment on Nurr1 expression in individual post-mitotic mesDAs (*F*_(1,40)_ = 14.451, *p* < 0.01; Fig. 4H). Supporting this, the distribution analysis revealed significant main effects of VIT_D_ treatment where more mesDAs had greater Nurr1 expression (*F*_(19, 684)_ = 6.243, *p* < 0.01; Fig. 4I).

#### TH Expression

There was a significant main effect of VIT_D_ treatment on TH expression in individual mature (Nurr1+TH+) mesDAs (*F*_(1,40)_ = 12.817, *p* < 0.01; Fig. 4K). Supporting this, the distribution analysis revealed significant main effects of VIT_D_ treatment where more mature mesDAs had greater TH expression (*F*_(19,684)_ = 5.301, *p* < 0.01; Fig. 4L).

## Discussion

### VITD abolishes MIA-induced sensitivity to AMPH

Rodent models of MIA and DVD-deficiency both exhibit early molecular and behavioural abnormalities consistent with altered DA signaling^6,7,10,35^, suggesting a common pathogenic pathway^6^. In support of this hypothesis, the present study shows that co-administration of VIT_D_ during gestation prevents the MIA-induced potentiation of AMPH sensitivity in adult offspring. It thus appears that the process leading to AMPH hypersensitivity in MIA offspring is initiated during early fetal development, such that interventions targeting the initiation process (e.g., through co-administration of VIT_D_ during gestation) are capable of preventing the subsequent establishment of this phenotype.

The failure of VIT_D_ to block MIA-induced impairments in PPI may seem counterintuitive at first, given that dopaminergic mechanisms have also been implicated in this association^11,36^. More specifically, previous studies found that acute and chronic administration of DA receptor antagonists in adulthood and adolescence, respectively, mitigated PPI deficits in adult MIA offspring^11,36^. While these findings clearly point to an involvement of the DA system in the adolescent and/or adult expression of PPI, they are not informative with regards to the underlying developmental processes occurring prior to adolescence. Based on the present data, we propose that MIA-induced alterations in PPI and AMPH sensitivity have a differential ontogeny. This hypothesis is also consistent with our previous findings showing that (i) whilst DVD-deficiency induces locomotor sensitivity to AMPH^13^, it does not impair PPI^12^, and (ii) MIA-induced PPI impairments are not critically dependent on the mesDA differentiation and maintenance factor, Nurr1^37^, the latter of which we find to be deregulated in the fetal brain after DVD-deficiency or MIA.

The acute elevation of inflammatory cytokines is proposed as one of the primary pathways leading to neuropsychiatric phenotypes in MIA offspring^35,38^. Given the well-known anti-inflammatory actions of vitamin D, this could well represent its neuroprotective mechanism^39^. However, we have previously reported that VIT_D_ co-treatment in this model failed to affect poly(I:C)-induced elevations in IL-6, IL-1ß and TNF alpha, in either maternal serum or fetal brain^27^. Although there may be other inflammatory factors/processes involved^40^, our current findings suggest an alternative neuroprotective mechanism. Based on our novel data presented here, we believe that maternal VIT_D_ may exert its beneficial effects through actions on the developing DA system.

### Both MIA and VITD decrease mesDA progenitor numbers, but only VITD increases mature mesDA number

All maternal treatments reduced mesDA progenitor (Lmx1a+Sox2+) numbers. There are likely to be distinct mechanisms for these effects, and consequences thereof as well. Whilst the MIA-induced reduction in mesDA progenitor cells was accompanied by the subsequent emergence of behavioral deficits, maternal VIT_D_ was without consequences on offspring behavior per se. Mechanistically, the effects of MIA on mesDA progenitor numbers likely involve the inhibition of neural progenitor formation via signaling at toll-like 3 receptors (TLR3), which are expressed early in the embryonic brain^41^. The pro-inflammatory cytokines induced by MIA can inhibit neural proliferation as well^42–44^.

Contrary to MIA, maternal VIT_D_ may reduce mesDA progenitor numbers by means of shifting the progenitor cells to a more differentiated phenotype. Indeed, the pro-differentiation properties of VIT_D_ in neurons and in the brain are well described. *In vitro* VIT_D_ treatment differentiates neural stem cells into neurons^45^; SH-SY5Y cells into DA neurons^21,22^ and inhibits neural progenitor formation^46^. In contrast, the *absence* of vitamin D during gestation leads to effects that are diametrically opposite to those reported here, namely an increase in mitosis across multiple brain regions^47,48^ and enhanced neural progenitor formation^46^.

Importantly, our study further shows that VIT_D_ and MIA have divergent effects on mature (Nurr1+TH+) mesDA neuron number. Although not significant, MIA clearly appeared to reduce (Nurr1 + TH+) mesDA cell number. In contrast, VIT_D_ increased (Nurr1+TH+) mesDA number, particularly for the more posterior mesDAs (Fig. 2L). A VIT_D_-mediated restoration of TH cell number therefore represents one plausible mechanism for the normalization of DA-mediated behaviours in MIA exposed offspring.

### VITD restores MIA-induced alterations in the distribution of mesDAs

For post-mitotic neurons, we show that MIA alters the mean DV positioning of post-mitotic (Lmx1a+Sox2−) mesDAs and the ML positioning of mature (Nurr1+TH+) mesDAs. VIT_D_ co-treatment did not rescue these deficits. However when examining the DV positioning of mature mesDAs, we show a profound MIA-induced reduction in DV positioning, which was fully prevented by VIT_D_. Importantly, VIT_D_ by itself had no effect on ML or DV positioning of any mesDA neuron type. Therefore, the correction of early mis-positioning of mesDA neurons by VIT_D_ could represent another plausible mechanism for the normalization of DA-mediated behaviours in MIA-exposed offspring.

### VITD regulates protein expression of key mesDA factors

VIT_D_ treatment increased the expression of all mesDA-related proteins examined. In contrast, MIA by itself had no significant effects on the expression of these proteins.

Here, we show that VIT_D_ treatment upregulates the expression of Lmx1a in developing mesDAs. Lmx1a is a convergent upstream molecule from SHH-Wnt pathways that specify mesDA lineage^49–51^. SHH and Wnt signaling are affected in MIA and DVD-deficiency models respectively^14,21^.

Nurr1 a direct upstream target of Lmx1a promotes mesDA differentiation^49,50,52^. MIA and DVD-deficiency reduce the mRNA expression of Nurr1 in the fetal brain^14,15^. This is consistent with present cell counting data, which indicates a recognizable (but not significant) reduction in Nurr1^+^ cell number in MIA embryos. At an individual cell level, however, the remaining cells have normal Nurr1 protein expression. Therefore, the likely explanation for previous findings showing reduced Nurr1 mRNA expression in MIA fetuses is a reduction in cell number^14^. Importantly, genetic deficiency of Nurr1 has previously been shown to induce AMPH hypersensitivity in a similar way to MIA^53^. Therefore the promotion of Nurr1 expression by VIT_D_ may represent a third mechanism for the prevention of AMPH hypersensitivity in MIA exposed offspring.

Lmx1a and Nurr1 regulate the expression of TH^54,55^. We have shown VIT_D_ elevates TH mRNA and protein in neuroblastomas in culture^21,22^. Again consistent with these findings the absence of VIT_D_ in DVD-deficiency leads to the downregulation of TH in fetal rodent brains^16,20^. Here, we verified that VIT_D_ increases the expression of TH *in vivo*.

In summary VIT_D_ increases the expression of the mesDA transcription factors (Lmx1a and Nurr1) and the rate-limiting enzyme in DA synthesis (TH) in developing mesDA cells. The expression of these three factors in control mesDAs clearly increases with differentiation state (Fig S4). Therefore VIT_D_ acts to differentiate the remaining mesDAs in MIA-exposed embryos. This represents a highly plausible mechanism for the prevention of DA-related behavioural deficits induced by MIA. Given that VIT_D_ treatment itself has no effect on the behaviours examined here and those examined previously^27^, we conclude that the accelerated maturation effects of VIT_D_ treatment alone on mesDAs is benign.

## Limitations

Although our findings provide more data for the translational relevance of introducing dietary intervention during early pregnancy for the prevention of developmental psychiatric diseases, the direct supplementation of the hormonal form of vitamin D, 1,25OHD, to pregnant women would adversely affect calcium in the developing embryo and therefore is not clinically feasible. Therefore preclinical replication studies are now warranted using the safe to use dietary form of vitamin D, cholecalciferol. In this study, we have only investigated one developmental time point and only male foetuses. In future studies, it will be obviously important to examine the entire ontogeny of mesDA systems in both sexes to understand long-term outcomes.

## Conclusion

This study represents a small but growing number of preclinical studies showing that the trajectory of adverse MIA-induced outcomes for adult brain function can be diminished or averted by dietary interventions. Two such studies have employed dietary interventions in MIA-exposed juvenile offspring with either a ketogenic diet or glucoraphanin to reverse certain MIA-induced behavioural phenotypes of relevance to psychiatry^56,57^. Of closer relevance to our study Weiser and colleagues have supplemented MIA dams with docosahexaenoic acid and prevented the onset of autistic-like phenotypes^58^. We recently reported that maternal treatment with VIT_D_ blocked the emergence of MIA-induced deficits in cognitive and social behavioural phenotypes of relevance to autism in offspring^27^. Using a mouse model of MIA, the present study is the first to reveal preventive effects of maternal VIT_D_ treatment on AMPH hypersensitivity, a phenotype with relevance to the positive symptoms of schizophrenia^59–61^.

We further identify three plausible pathways via which VIT_D_ may act to restore or support normal DA development in the event of MIA. First, MIA retarded the positioning of mesDAs particularly in the DV dimension, whereas VIT_D_ treatment restored this at least for TH+ mesDA neurons. Second, MIA appeared to reduce TH+ cell number, while at the same time, VIT_D_ increased TH+ mesDA cells. Third, VIT_D_ increased the expression of proteins consistent with a more differentiated mesDA neuron. These three processes likely reflect an accelerated differentiation of DA neurons induced by VIT_D_, which in turn may have protected against the emergence of AMPH hypersensitivity in adult MIA offspring. By contrast, MIA-induced PPI deficiency does not seem to critically involve the same developmental pathways.

This data adds to a steadily increasing literature indicating developing DA systems may be particularly vulnerable to adverse environmental factors such as MIA and DVD-deficiency and represent a plausible convergent early mechanistic pathway to psychiatric disorders such as schizophrenia.

## Electronic supplementary material

Supplemental info
