# Supplementary material for: Maternal Vitamin D Prevents Abnormal Dopaminergic Development and Function in a Mouse Model of Prenatal Immune Activation
Source: Sci Rep. 2018 Jun 27;8:9741. doi: 10.1038/s41598-018-28090-w (PMC6021387; doi:10.1038/s41598-018-28090-w)
Supplement: Supplementary file 1 — Supplemental info [file 41598_2018_28090_MOESM1_ESM.pdf]

Title:

Maternal Vitamin D Prevents Abnormal Dopaminergic Development and Function in a Mouse Model of Prenatal Immune Activation

Wei Luan[1], Luke Alexander Hammond[2], Stephanie Vuillermot[3], Urs Meyer[4,5], Darryl Walter Eyles[1,6]*

1. Queensland Brain Institute, The University of Queensland, Brisbane, QLD, Australia.
2. Zuckerman Mind Brain Behavior Institute, Columbia University, New York, New York, USA.
3. Physiology and Behavior Laboratory, ETH Zurich, Schwerzenbach, Switzerland.
4. Institute of Pharmacology and Toxicology, University of Zurich-Vetsuisse, Zurich, Switzerland.
5. Neuroscience Centre Zurich, University of Zurich and ETH Zurich, Zurich, Switzerland.
6. Queensland Centre for Mental Health Research, Brisbane, QLD, Australia.

*Correspondence to:

Darryl Walter Eyles, PhD

Queensland Brain Institute

University of Queensland

Brisbane QLD 4072 Australia

Phone: +61 7 3346 6370

Fax: +61 7 33466301

Email: eyles@uq.edu.au

SUPPLEMENT METHODS

Prepulse Inhibition of the Acoustic Startle Reflex for POL-VIT$_D$ co-administration experiment

Briefly a session began with the animals being placed into the plexiglas enclosure. Animals were acclimatized to the apparatus for 2 min prior to the first trial. The first six trials consisted of six startle-alone trials, comprising two trials of each of the three possible pulse intensities. These trials served to habituate and stabilize the animals' startle response and were not included in the analysis. Subsequently, the animals were presented with 10 blocks of discrete test trials. Each block consisted of the following: three pulse-alone trials (100, 110, or 120 dB$_A$), three prepulse-alone trials (+6, +12, or +18 dB$_A$ above background), nine possible combinations of prepulse-plus-pulse trials (3 levels of prepulse × 3 levels of prepulse), and one no-stimulus trial. The 16 discrete trials within each block were presented in a pseudorandom order, with a variable interatrial interval of a mean of 15 s (ranging from 10 to 20 s). For each of the three pulse intensities (100, 110, or 120 dBA), PPI was indexed by percentage inhibition of the startle response obtained in the pulse-alone trials by the following expression: 100% x (1 - [mean reactivity on prepulse-plus- pulse trials/mean reactivity on pulse-alone trials]), for each subject, and at each of the three possible prepulse intensities (+6, +12, or +18 dB$_A$ above background). In addition, the animals' reactivity to pulse-alone trials (i.e., startle reactivity) and prepulse-alone trials (i.e., prepulse-induced reactivity) were also measured and analyzed as previously described[1,2].

Image processing and quantitative immunochemistry

To assess Lmx1a+ and/or Sox2+ mesDAs a mask channel to facilitate object segmentation was first created using ImageJ/Fiji. This mask channel combined Lmx1a and Sox2 channels and was processed using a sequence of background subtraction (rolling ball), removal of outliers,

and local contrast enhancement. The mask channel and original channel files for each section were subsequently processed with a pipeline developed in CellProfiler (Broad Institute, MA, USA) [3]. This pipeline used the mask channel for the detection of all Lmx1a+ and/or Sox2+ nuclear objects, which were then further filtered into subpopulations according to the mean fluorescence intensity measured on original channels (Lmx1a: threshold > 0.0107, Sox2: threshold > 0.0045).

To assess Nurr1 and TH expressing cells mask channels for both channels were first created using ImageJ/Fiji to facilitate object segmentation, as described above. These mask channels and the original channel files for each section were subsequently processed with a pipeline developed in CellProfiler. This pipeline first segmented Nurr1+ nuclear objects using the Nurr1 mask channel. These objects where then used as seeds for region growing to detect TH positive cytosol using the TH mask channel. The Nurr1 nuclear mask and TH cytosolic masks were then used to determine the mean fluorescence intensity of their respective original channels (Nurr1: threshold > 0.002, TH: threshold > 0.006). Cells were split into two subpopulations, mature mesDAs (Nurr1+TH+) and immature mesDAs (Nurr1+TH-).

The following single-cell measurements were conducted for mesDA subgroups using CellProfiler software: cell number (= the number of identified object); the size of nuclei or cytosolic region (μm^2); nuclear morphologies including eccentricity (calculated as the ratio of the distance between the foci of object and its maximal axis length), the maximal or minimal axis length (calculated as the length of the maximal or minimal axis of the nuclei); the mean fluorescence intensity of protein of interests including Sox2, Lmx1a, Nurr1 and TH. In addition, the coronal positioning of mesDA cells were measured according to the center of mesDA nuclei (x_n, y_n) relative to the most ventral point of the ventricle along the midline, the coordinates (x_0, y_0). Therefore, the mediolateral (ML) positioning (x) of individual nuclei of mesDAs to the

midline was calculated as (x_n - x_0). The dorsoventral (DV) position (y) of individual nuclei of mesDAs was calculated as (y_n - y_0).

Accuracy check on CellProfiler identification

The accuracy of CellProfiler identification of mesDA cells were manually counted by W.L based on the output images of CellProfiler software with the group-code blinded. The identification accuracy (%) was calculated = (correct identified objects) *100/(correct identified objects + false positively identified objects + false negatively identified objects + oversegmented objects + undersegmented objects) (Fig S2).

SUPPLEMENT RESULTS

VIT_D dose (400ng/kg/2ml) showed no effects on AMPH-mediated locomotion or PPI

There were no obvious significant effects of maternal VIT_D administration on the PPI of adult offspring under all examined conditions (*p's* > 0.05) or the mean PPI% (*p* > 0.05; Fig S1A). There were no noticeable effects of maternal VIT_D treatment on locomotor activities of adult offspring during the habituation or post-AMPH treatment phase (*p's* >0.05; Fig S1B).

Accuracy check on CellProfiler

The CellProfiler pipeline provided an accuracy of 87.54 + 3.39 % (ranging 76.34 - 93.65%) for identification of mesDA progenitors (Lmx1a+Sox2+) among treatment groups (Fig S2B), and accuracy of 90.09 + 3.21 % (ranging 84.54 – 96.83 %) for post-mitotic (Lmx1a+Sox2-) mesDAs (Fig S2C), respectively. Accuracy was not affected by treatment (*p's* > 0.05; Fig S2B and S2C).

The CellProfiler pipeline provide an overall average accuracy of 80.07 + 5.03 % (ranging 72.72 - 93.61%) for Nurr1+ mesDAs identification (Fig S2E), and an overall average accuracy of 75.98 + 4.03 % (ranging 67.59 – 86.89%) for TH+ mesDAs identification (Fig S2F). No significant differences of identification accuracy for Nurr1+ mesDAs were noticeable among treatment groups (*p's* > 0.05). There were no significant differences of identification accuracy for post-mitotic mesDAs among treatment groups (*p's* > 0.05).

Cellular morphologies were not altered by prenatal treatments

We investigated the nuclear size, cytosolic size, and eccentricity of mesDAs. However, there were no significant effects of MIA or VIT_D treatments on these morphological parameters (*p's* > 0.05; Fig S3). However, there were significant differences of these morphological

parameters between mesDA progenitors (Lmx1a+Sox2+) and post-mitotic (Lmx1a+Sox2-) mesDA cells. mesDA progenitors (Lmx1a+Sox2+) show larger nuclear area than post-mitotic (Lmx1a+Sox2-) mesDAs ($p < 0.0001$; Fig S3A). The mean nuclear eccentricity of mesDA progenitors (Lmx1a+Sox2+) was larger than of post-mitotic (Lmx1a+Sox2-) mesDA cells ($p < 0.0001$; Fig S3D). In addition, mesDA progenitors (Lmx1a+Sox2+) also show longer maximal axis length than post-mitotic (Lmx1a+Sox2-) mesDAs ($p < 0.0001$; Fig S3E). The minimal axis length of progenitors was also found significantly longer than of post-mitotic mesDAs ($p < 0.01$; Fig S3F).

Increased protein expression of Lmx1a and Nurr1 in differentiated mesDAs

We observed main effects of the stage of cell maturation on the expression of Lmx1a and Nurr1 in control embryos (CON-VEH) (Fig S4). The mean intensity of Lmx1a was higher in post-mitotic (Lmx1a+Sox2-) mesDAs than in mesDA progenitors (Lmx1a+Sox2+) (paired t-test, t=10.61, $p < 0.0001$; Fig S4A). A representative coronal medial MES section (Fig S4B) showing the differential expression of Lmx1a is visualized in a heat map image (Fig S4C). In post-mitotic mesDA subpopulations, the mean intensity of Nurr1 was significant greater in the mature (Nurr1+TH+) mesDAs compared with immature (Nurr1+TH-) mesDAs (paired t-test, t=9.409, $p < 0.001$; Fig S4D). Similarly, a representative coronal medial MES section (Fig S4E) showing the differential expression of Nurr1 is visualized in a heat map image (Fig S4F). To us this suggests that the upregulation of all three afore-mentioned proteins in VIT_D-treated mesDAs represents an increase in the differentiation status of these neurons at GD11.

SUPPLEMENTAL FIGURE LEGENDS

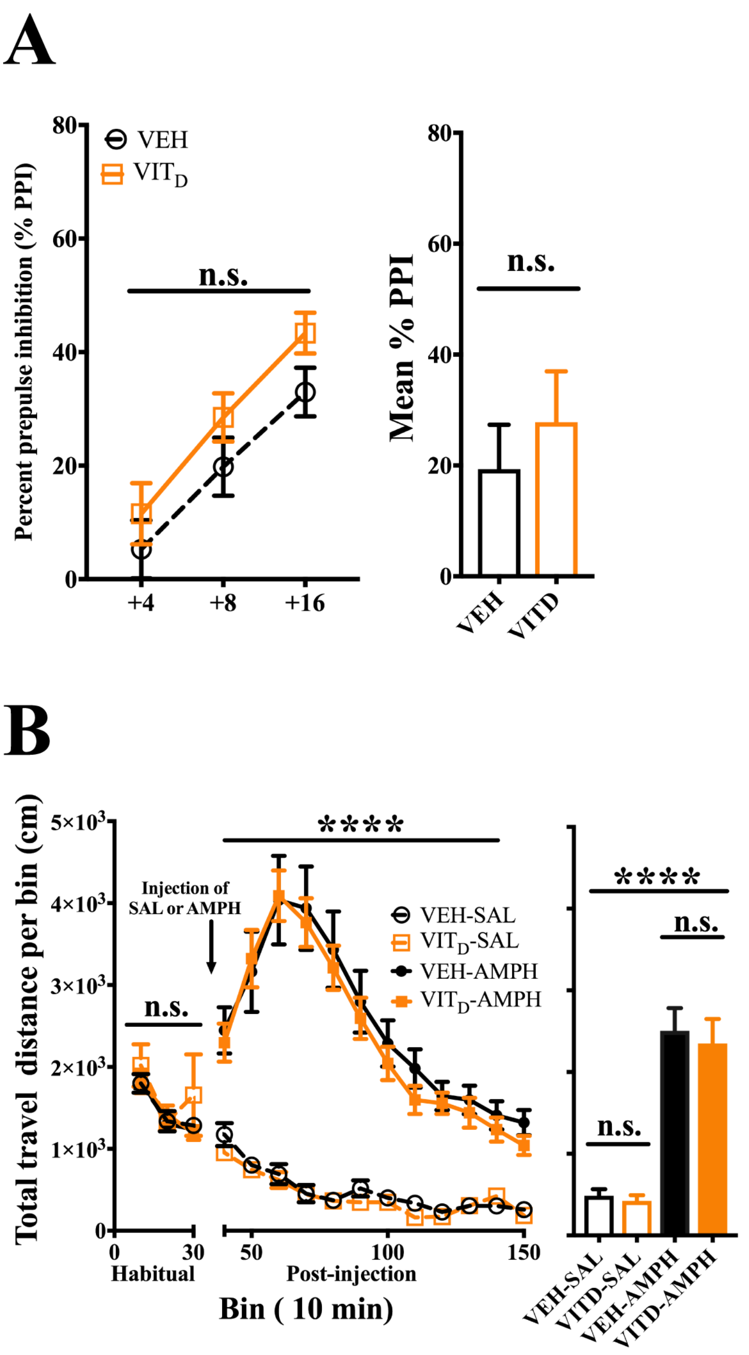

Figure S1. Maternal VIT_D treatment did not alter dopamine (DA)-regulated behaviours in offspring.

(A) Maternal VIT_D treatment does not alter sensorimotor gating deficits in adult offspring (PND70). Prepulse inhibition (PPI) of the acoustic startle reflex was used to investigate the sensorimotor gating in postnatal offspring. The line plots show %PPI as a measure of the three

intensities of prepulse corresponding to +4, +8 and +16 dB_A above the background white noise respectively. The bar plots represent the mean %PPI for all prepulse and pules levels. All values were medians ± SEM.

(B) Adult offspring born to vehicle (corn oil, VEH) or VIT_D (1,25OHD)-treated dams received amphetamine (AMPH) at and their locomotor behaviors were assessed in an open field arena. There were no significant differences in basal locomotor activity and following saline (SAL)/AMPH administration treated VIT_D compared to VEH ($p > 0.05$). As expected, AMPH treatment resulted in a significant increase in locomotor activity in both VEH- or VIT_D-treated offspring compared to saline treatment ($p < 0.05$). Bar plots represent the mean travel distance of tested offspring after AMPH administration. All values were means ± SEM.

VEH: N = 8; VIT_D: N = 9. * $p < 0.05$, **** $p < 0.001$, n.s., not statistically significant.

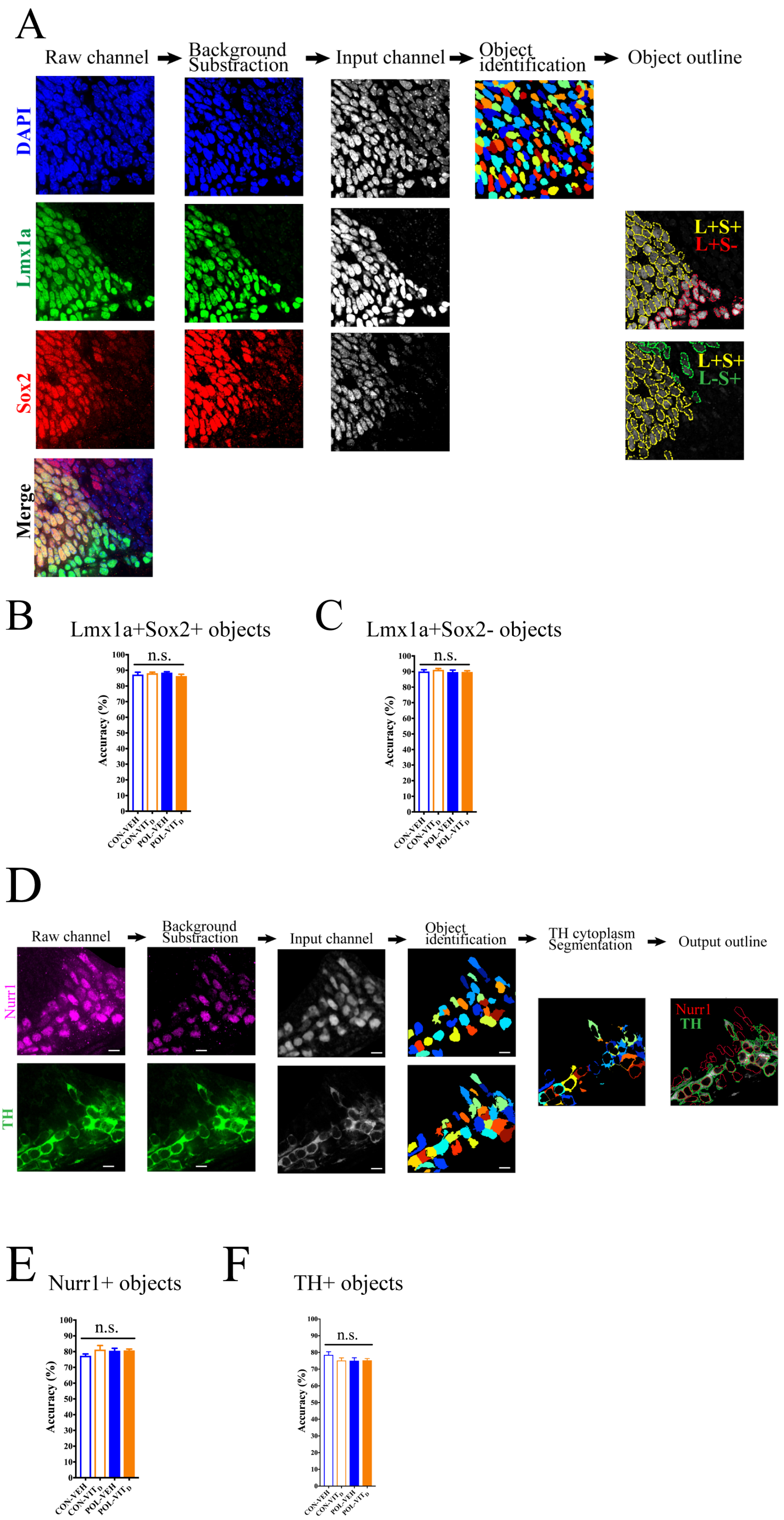
A
Raw channel
Background Substraction
Input channel
Object identification
Object outline
DAPI
Lmx1a
Sox2
Merge
L+S+
L+S-
L+S+
L-S+
B
Lmx1a+Sox2+ objects
C
Lmx1a+Sox2- objects
n.s.
Accuracy (%)
CON-VEH
CON-VIT$_D$
POL-VEH
POL-VIT$_D$
D
Raw channel
Background Substraction
Input channel
Object identification
TH cytoplasm Segmentation
Output outline
Nurr1
TH
E
Nurr1+ objects
F
TH+ objects

Figure S2. The image analysis pipeline and accuracy check for identification of mesencephalic dopaminergic (mesDA) cells in gestational day (GD) 11 fetal brain using ImageJ/CellProfiler.

(A) The ImageJ/ CellProfiler pipeline for segmentation of Lmx1a+ developing mesDA cells in the floor plate (FP) of coronal mesencephalic (MES) sections. Coronal MES sections are triple-labeled with DAPI, Lmx1a (green) and Sox2 (red). First, an additional mask channel to facilitate object segmentation is created in ImageJ/Fiji using a sequence of background subtraction. Next, CellProfiler is used to automatically segment individual nuclei and measure the mean intensity of the original Lmx1a and Sox2 channels, nuclei position and morphological measurements. Segmented nuclei are subsequently filtered according to the mean fluorescence intensities of Lmx1a or Sox2 per nucleus. Images revealing the outlines of detected nuclei are used for validation, here the mesDA progenitors (Lmx1a+Sox2+) are indicated by yellow lines. Post-mitotic mesDAs (Lmx1a+Sox2-) are outlined with red lines. Non-mesDA cells are outlined with green lines. L, Lmx1a; S, Sox2.

(B) Overall accuracy for identification of mesDA progenitors (Lmx1a+Sox2+). There were no significant differences of accuracy among treatment groups (p's >0.05).

(C) Overall accuracy for post-mitotic (Lmx1a+Sox2-) mesDAs. There were no significant differences of accuracy among treatment groups (p's >0.05).

(D) The ImageJ/CellProfiler pipeline for segmentation of Nurr1+ post-mitotic mesDAs in the FP of coronal MES sections. Coronal MES sections are labeled by nuclear Nurr1 (magenta) and cytosolic TH (green). Original channel images are processed in ImageJ to create mask channels for improved cell segmentation as previously described. The enhanced mask channels are used by CellProfiler to first segment individual Nurr1 positive nuclei and subsequently detect TH in the cytosol via region growing. Nurr1+ post-mitotic neurons are segmented and

identified according to the mean intensity of Nurr1+. Subsequently mature (Nurr1+TH+) mesDAs are filtered according to the mean intensity of TH.

(E) Overall accuracy for Nurr1+ mesDAs identification. There were no significant differences of accuracy among treatment groups (p’s >0.05).

(F) Overall accuracy for TH+ mesDAs identification. There were no significant differences of accuracy among treatment groups (p’s >0.05).

All values were means + SEM. n.s. represents not statistically significant.

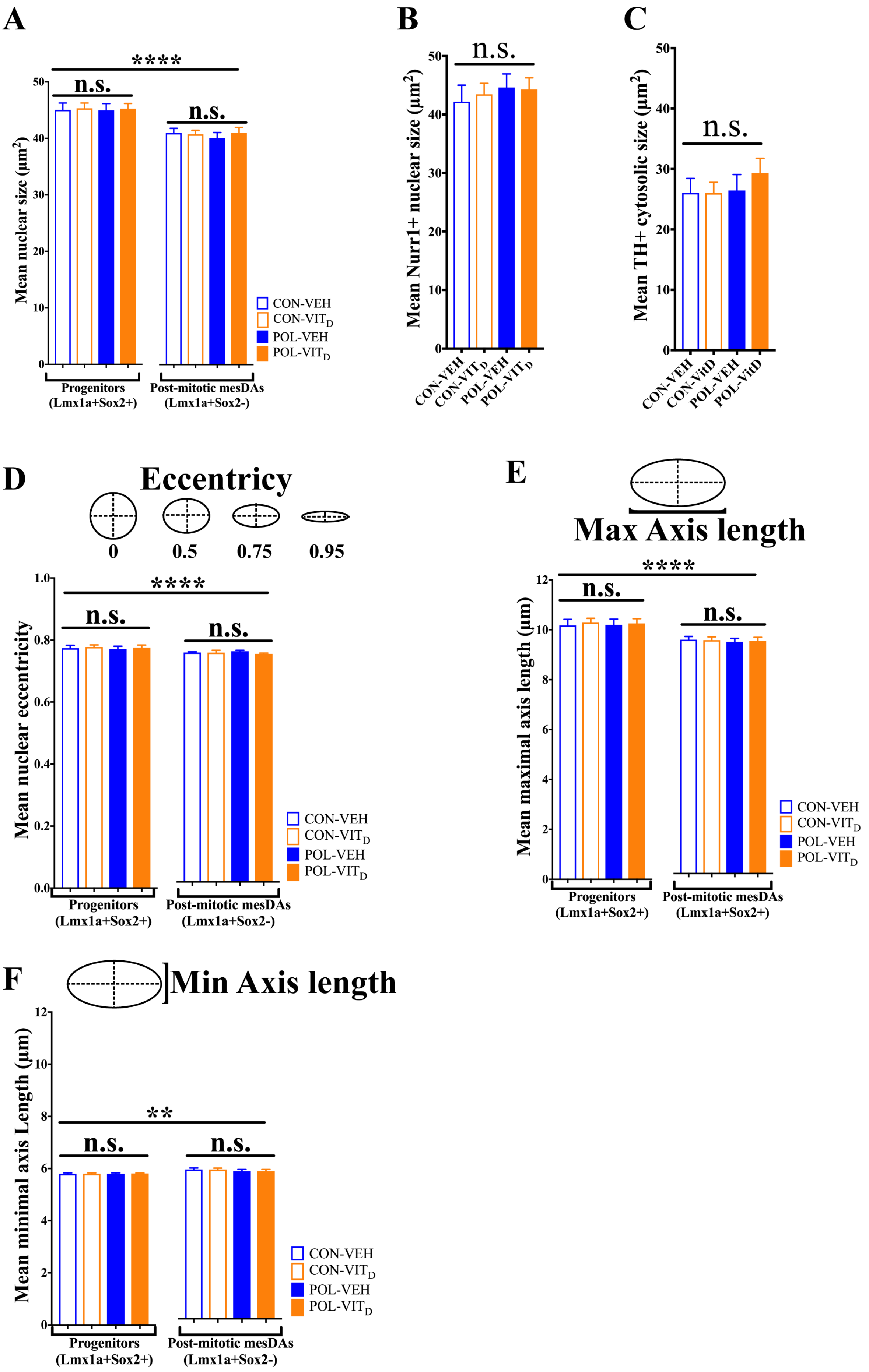
A
B
C
****
n.s.
Mean nuclear size (µm²)
Progenitors (Lmx1a+Sox2+)
Post-mitotic mesDAs (Lmx1a+Sox2-)
CON-VEH
CON-VIT_D
POL-VEH
POL-VIT_D
Mean Nurr1+ nuclear size (µm²)
Mean TH+ cytosolic size (µm²)
CON-VitD
POL-VitD
D
Eccentricity
0
0.5
0.75
0.95
Mean nuclear eccentricity
E
Max Axis length
Mean maximal axis length (µm)
Post-mitotic mesDAs (Lmx1a+Sox2+)
F
Min Axis length
**
Mean minimal axis Length (µm)

Figure S3. MIA or VIT$_D$ treatment did not alter cellular morphologies of developing mesencephalic dopaminergic (mesDA) cells.

There were no significant differences in the mean nuclear size (μm_2) of (A) mesDA progenitors (Lmx1a+Sox2+) and of post-mitotic (Lmx1a+Sox2-) mesDAs, and (B) post-mitotic (Nurr1+) mesDAs among the prenatal treatment groups (*p's* > 0.05). However, mesDA progenitors (Lmx1a+Sox2+) show larger mean nuclear size than post-mitotic (Lmx1a+Sox2-) mesDAs ($p < 0.0001$).

(C) Prenatal treatments did not significantly alter the TH+ average cytosolic area in Nurr1+TH+ mesDAs (*p's* > 0.05).

(D) There were no significant differences of nuclear eccentricity in Lmx1a+ progenitors among treatment groups (*p's* >0.05). Mean nuclear eccentricity of progenitors was larger than of post-mitotic cells ($p < 0.0001$).

(E) MIA or VIT$_D$ treatments did not significantly affect the maximal axis length Lmx1a+ progenitors among groups ($p > 0.05$). mesDA progenitors (Lmx1a+Sox2+) also show longer maximal axis length than post-mitotic (Lmx1a+Sox2-) mesDAs ($p < 0.0001$).

(F) There were no significant effects of MIA or VIT$_D$ treatments on the minimal axis length of progenitors (Lmx1a+Sox-) among treatment groups (*p's* > 0.05). The minimal axis length of progenitors was also found significantly longer than of post-mitotic mesDAs ($p < 0.01$).

All values were means ± SEM. ** $p < 0.01$, **** $p < 0.0001$, n.s. represents not statistically significant.

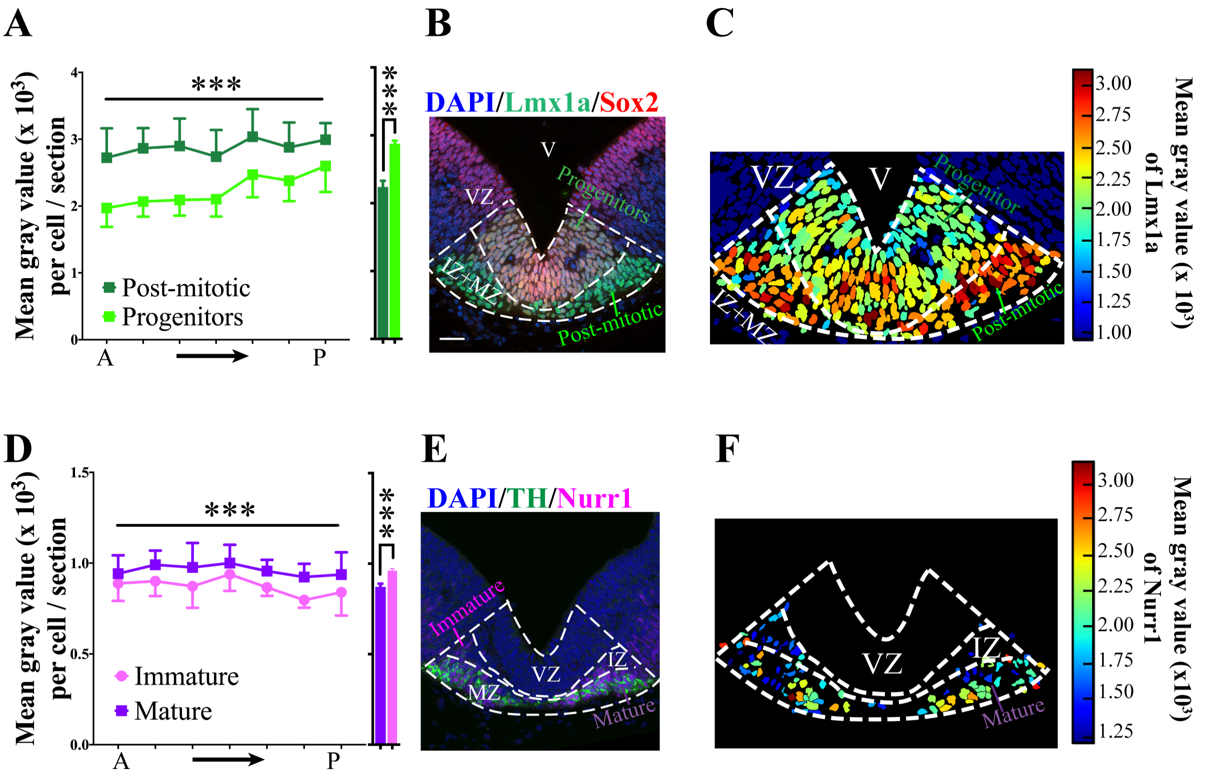

Figure S4. the expression of Lmx1a and Nurr1 increase in mesencephalic dopamine neurons (mesDAs) increase with differentiation.

(A) Plots representing the mean gray value of Lmx1a in all examined mesDA cells in the floor plate (FP) of coronal mesencephalic (MES) sections along the anterior-posterior (A-P) axis. The mean intensity of Lmx1a was significantly higher in post-mitotic (Lmx1a+Sox2-) mesDAs than in mesDA progenitors (Lmx1a+Sox2+) ($p < 0.001$). Bar plots represent the average mean intensity of Lmx1a between mesDA subgroups.

(B) A representative triple-labeled image of mesDA cells in the FP of medial coronal MES section using DAPI (blue), Lmx1a (green) and Sox2 (red). White dash lines outline ventricular (VZ), intermediate zone (IZ) and mantle zone (MZ) were delineated by dash lines.

(C) A heat map image shows the increased expression of Lmx1a in post-mitotic IZ and MZ compared with progenitors restricted largely to the VZ as quantified in (B). Bar plot represents a spectrum of colors coding the mean intensity of Lmx1a from low to high in a range of 0.016-0.048.

(D) Plots representing the mean gray value of Nurr1 in mesDA cells in the FP of coronal MES sections along A-P axis. The mean intensity of Lmx1a was significantly higher in mature (Nurr1+TH+) mesDAs than in immature (Nurr1+TH-) mesDAs ($p < 0.01$). Bar plots represent the average value of Nurr1 protein levels between mesDA subgroups.

(E) A representative image of triple-labeled post-mitotic mesDAs in FP of coronal MES sections using DAPI (blue), TH (green) and Nurr1 (magenta). VZ and (IZ+MZ) layers were delineated by white dash lines.

(F) A heat map image shows the increased expression of Nurr1 in mature (Nurr1+TH+) mesDAs in the MZ compared to immature (Nurr1+TH-) mesDAs in the IZ. Bar plot represents a spectrum of colors coding the mean intensity of Nurr1 from low to high in a range of 0.020-0.034.

All values were means $\pm$ SEM. ** $p < 0.01$, *** $p < 0.001$, n.s. represents not statistically significant. Scale bars: 50 µm.

SUPPLEMENT REFERENCES

1 Meyer, U., Feldon, J., Schedlowski, M. & Yee, B. K. Towards an immuno-precipitated neurodevelopmental animal model of schizophrenia. *Neuroscience and biobehavioral reviews* **29**, 913-947, doi:10.1016/j.neubiorev.2004.10.012 (2005).

2 Meyer, U. *et al.* The time of prenatal immune challenge determines the specificity of inflammation-mediated brain and behavioral pathology. *The Journal of neuroscience : the official journal of the Society for Neuroscience* **26**, 4752-4762, doi:10.1523/JNEUROSCI.0099-06.2006 (2006).

3 Vokes, M. S. & Carpenter, A. E. Using CellProfiler for automatic identification and measurement of biological objects in images. *Curr Protoc Mol Biol* **Chapter 14**, Unit 14 17, doi:10.1002/0471142727.mb1417s82 (2008).
